# Accurate weed detection in UAV grassland images via intelligent annotation and multi-dimensional network enhancement

**DOI:** 10.3389/fpls.2026.1872976

**Published:** 2026-07-01

**Authors:** Qiang Wang, Ruihan Bai, Chunxiao Wei, Ziyi Deng

**Affiliations:** 1 UAV Industry Academy, Chengdu Aeronautic Polytechnic University, Chengdu, China; 2 School of Civil and Transportation Engineering, Hohai University, Nanjing, China

**Keywords:** intelligent annotation, unmanned aerial vehicle images, vision-language model, weed detection, YOLOv11

## Abstract

**Introduction:**

Accurate weed detection from unmanned aerial vehicle (UAV) images remains difficult because weed targets often blend with surrounding vegetation, vary substantially in scale, and exhibit irregular boundaries under cluttered grassland backgrounds.

**Methods:**

This study presents a UAV grassland weed detection framework that combines intelligent annotation with task-oriented detector adaptation. For label generation, SAM produces instance-level vegetation masks, SigLIP performs few-shot semantic matching, and a vision-language model audits ambiguous candidates using few-shot weed references, local candidate patches, and full-image context. Candidate outputs are converted into YOLO-format labels for detector training. For detection, a YOLOv11-based model is adapted with four complementary components: EIEStem for shallow boundary preservation, C3k2-EMA for multi-scale feature aggregation, SPPF-LSKA for contextual modeling, and LDConv for adaptive downsampling of irregular weed targets.

**Results:**

On 200 reference images, the SAM–SigLIP–VLM workflow achieved 92.4% label precision, a mean IoU of 0.813, and an F1-score of 0.905, while reducing the annotation time per image from 96.8 s to 31.6 s. Under five-fold image-level cross-validation on a UAV grassland weed dataset, the improved detector achieved 0.762 ± 0.004 mAP@0.5 and 0.545 ± 0.005 mAP@0.5:0.95, improving the YOLOv11 baseline by 4.0 and 4.7 percentage points, respectively.

**Discussion:**

Additional evaluations cover annotation quality, module ablation, module placement, downsampling mechanisms, detector comparison, and validation on an external public crop–weed dataset. These results show that the proposed framework improves both annotation efficiency and UAV grassland weed detection performance in complex vegetation backgrounds.

## 1 Introduction

Accurate weed detection is an important basis for site-specific weed management and precision herbicide application. Conventional weed control still depends largely on manual field inspection and broad-area herbicide spraying. These practices are labor-intensive, inefficient, and may increase chemical use and environmental risk (Gerhards et al., 2022). UAV images provide a flexible way to observe grassland scenes at high spatial resolution. When combined with deep learning, they offer a promising solution for automatic weed detection over large areas (Kamilaris and Prenafeta-Boldú, 2018; Sandoval-Pillajo et al., 2025).

However, UAV-based weed detection remains difficult in practical grassland scenes. Weeds often share similar color and texture with surrounding vegetation, and their appearance changes with growth stage, illumination, and imaging height. In addition, many weed instances have irregular boundaries and small local structures. These factors make both label generation and model learning more difficult. In dense vegetation scenes, manual annotation is slow and inconsistent, while standard object detectors may miss weak weed cues or confuse weeds with background plants.

An annotation workflow based on SAM (Kirillov et al., 2023), SigLIP (Zhai et al., 2023), and a vision-language model (VLM) (Li et al., 2023) is used to generate training labels. In this study, SigLIP is used to filter weed-like regions through few-shot semantic matching. SAM produces vegetation instance candidates, and the VLM checks difficult cases using few-shot weed references, local candidate patches, and full-image context. Candidate outputs are converted into YOLO-format labels for model training.

For the detection model, this study adapts YOLOv11 to UAV-view weed detection. General object detectors, including the YOLO series (Redmon et al., 2016; Hidayatullah et al., 2025), are efficient but are not specifically optimized for irregular weed targets in cluttered grassland backgrounds. During feature extraction and downsampling, shallow boundary details and small geometric cues may be weakened. To address this, detector adaptation combines boundary preservation, feature aggregation, context modeling, and adaptive downsampling in a unified YOLOv11-based architecture.

The main contributions are as follows.

First, a SAM–SigLIP–VLM-assisted annotation workflow is developed for UAV-view grassland weed images. It integrates instance mask generation, few-shot semantic matching, VLM-based auditing, manual verification, and YOLO-format label conversion.
Second, a YOLOv11-based detector is adapted for UAV grassland weed detection by enhancing four key feature stages: shallow boundary extraction, multi-scale feature fusion, high-level contextual modeling, and adaptive downsampling. This design targets the main visual difficulties of grassland weeds, including weak boundaries, scale variation, background confusion, and irregular plant structures.
Third, the complementary roles of the four detector components are analyzed. EIEStem enhances low-level boundary cues, C3k2-EMA refines multi-scale feature aggregation, SPPF-LSKA provides larger-context representation, and LDConv preserves irregular weed structures during downsampling. These components form a multi-level feature-enhancement chain across shallow, fusion, semantic, and downsampling stages.
Fourth, the framework is evaluated through annotation-quality analysis, progressive ablation, placement analysis, downsampling comparison, complexity-aware detector comparison, and additional validation on an external public crop–weed dataset. Repeated runs and cross-validation are used to report mean ± standard deviation for the main experiments.

## 2 Related work

### 2.1 Deep learning for UAV weed detection

Deep learning has become the main approach for agricultural weed detection. Earlier studies often used two-stage detectors, such as Faster R-CNN and FPN-based models, because of their strong localization ability (Saleem et al., 2022; Mu et al., 2022). These methods can provide accurate boxes, but their computational cost limits their use in UAV-based field applications. One-stage detectors, especially the YOLO family, have therefore become more common in agricultural detection because they offer a better balance between accuracy and speed (Redmon et al., 2016; Hidayatullah et al., 2025; Rahman et al., 2023; Liu et al., 2024).

Recent studies have further adapted YOLO models for weed detection. Yang et al. proposed GTDRYOLOv12 for complex agricultural weed scenes, Yue and Zhao improved YOLOv11-seg for UAV soybean weed segmentation, Chinnasami et al. combined YOLO11 with PSPNet for UAV-based weed analysis, and Peng et al. developed AGRI-YOLO for corn weed detection (Yang et al., 2025; Yue and Zhao, 2026; Chinnasami et al., 2025; Peng et al., 2025). These works show that weed detection benefits from detector designs matched to agricultural image characteristics.

Grassland scenes bring a different set of visual challenges from many row-crop weed scenarios. UAV images of grassland usually contain dense and continuous vegetation, with no clear crop-row structure to guide the detector. Weed targets are often small, irregular, weakly separated from the background, and visually similar to neighboring plants. For this reason, the YOLOv11-based detector in this study is adapted at four feature stages: early boundary extraction, multi-scale feature fusion in the neck, high-level contextual modeling, and detail-preserving downsampling. This detector design is combined with the SAM–SigLIP–VLM annotation workflow, so the framework covers both label generation and detector training for UAV grassland weed detection.

### 2.2 Public weed datasets and application gaps

Public weed datasets provide useful benchmarks, but they are not identical in imaging view, sensor type, annotation form, or target definition. WeedMap provides UAV multispectral data for crop–weed semantic mapping and is mainly used for segmentation evaluation (Sa et al., 2018). CoFly-WeedDB contains UAV RGB images from cotton fields with weed annotations, but the dataset is relatively small and is commonly used for mask-based segmentation or species identification (Krestenitis et al., 2022). USU-Corn-WeedDB is closer to box-level UAV weed detection because it provides RGB drone images and bounding-box labels in forage-corn fields (Bhandari et al., 2026).

Other weed datasets are also useful but serve different purposes. DeepWeeds contains *in-situ* rangeland weed images and mainly supports species-level classification rather than dense UAV object detection (Olsen et al., 2019). CropAndWeed provides bounding boxes, semantic masks, and stem positions for crop and weed plant instances, so it is suitable for detection-oriented external evaluation, although its viewpoint is different from UAV grassland imagery (Steininger et al., 2023). Overall, these public datasets provide useful references, but none of them fully matches the UAV grassland setting studied here. This motivates the construction of task-specific weed annotations and a detector adapted to dense grassland backgrounds.

### 2.3 Vision-language models and assisted annotation

Reducing annotation dependence is important for scalable weed detection. Transfer-learning and zeroshot studies show that pretrained visual representations can help when target-domain labels are limited. Belissent et al. investigated transfer and zero-shot learning for UAV weed detection and classification (Belissent et al., 2024). Recent agricultural vision-language datasets and benchmarks, such as VL-PAW and AgEval, further show that multimodal models can use visual examples and text descriptions for agricultural recognition and reasoning tasks (Yu et al., 2025; Arshad et al., 2025).

For UAV weed scenes, VLMs have also been explored for zero-shot weed detection, localization, and visual reasoning (Nasir et al., 2026). However, direct VLM-based detection is still difficult because small weeds may be missed and bounding boxes can be unstable. In this study, the VLM is therefore not used as a standalone detector. SAM first generates candidate regions, SigLIP compares candidate patches with a 10-shot weed support set, and Qwen3-VL-32B checks ambiguous candidates using support patches, the local candidate crop, and the full UAV image. This workflow uses the VLM for semantic checking, while candidate localization is provided by SAM regions and the final labels are verified before detector training.

## 3 Methods

Our method contains two coupled components: an intelligent annotation workflow for generating weed labels from UAV grassland images and an improved YOLOv11-based detector. The dataset setting is introduced first, followed by the annotation workflow and detector architecture.

### 3.1 Dataset description and task definition

Experiments were conducted on 2,360 UAV grassland images with 8,742 YOLO-format weed boxes. Each box belongs to a single target class, “weed”. Basic dataset information is listed in **Table 1**.

**Table 1:** Dataset metadata.

Item	Description
Dataset source	UAV grassland weed dataset collected in this study.
Number of images	2,360.
Image resolution	1920 × 1080 pixels after image extraction/cropping.
UAV platform	DJI Phantom 4 RTK.
Sensor/camera	1-inch 20 MP RGB CMOS camera.
Flight altitude	30–60 m above ground level.
Ground sampling distance	approximately 1.2–2.0 cm/pixel.
Illumination/weather conditions	natural outdoor illumination under sunny to cloudy conditions; image acquisition was not conducted during rainfall.
Weed species information	mixed grassland weeds, including broadleaf and grass-like weeds; species-level labels were not assigned in this

### 3.2 Intelligent instance segmentation annotation system based on multi-modal large models

**Figure 1:**
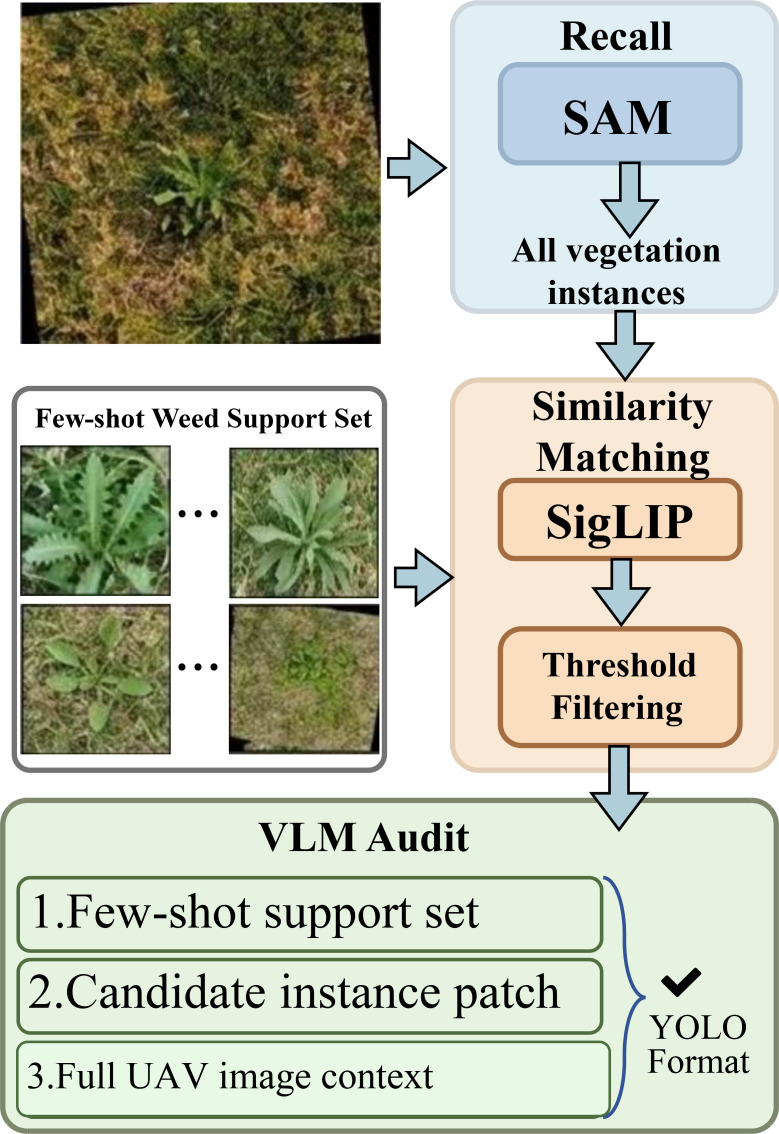
The overall framework of the intelligent instance segmentation annotation system.

**Figure 1** illustrates the intelligent instance segmentation and annotation system used for grassland weed detection. Segmentation, matching, and auditing form the three main stages of the workflow.

First, SAM (Kirillov et al., 2023) segments grassland images and generates instance masks for individual vegetation regions. A few-shot support set with 10 representative weed patches serves as the visual semantic reference. SigLIP (Zhai et al., 2023) computes cosine similarity between each segmented instance and the support samples, and candidates below the similarity threshold are removed as background vegetation.

Finally, a vision-language model (VLM) (Li et al., 2023) audits ambiguous candidates with three visual inputs: the 10 few-shot weed support patches, the cropped candidate patch, and the full UAV image that provides global scene context. The system prompt instructs the VLM to compare the local candidate with the support patches, use the full image to check scene consistency and boundary context, and then return a structured weed/non-weed judgment. Candidates marked as uncertain are passed to manual verification. Retained candidates are converted into normalized YOLO-format boxes before detector training.

### 3.3 Overall network architecture

**Figure 2:**
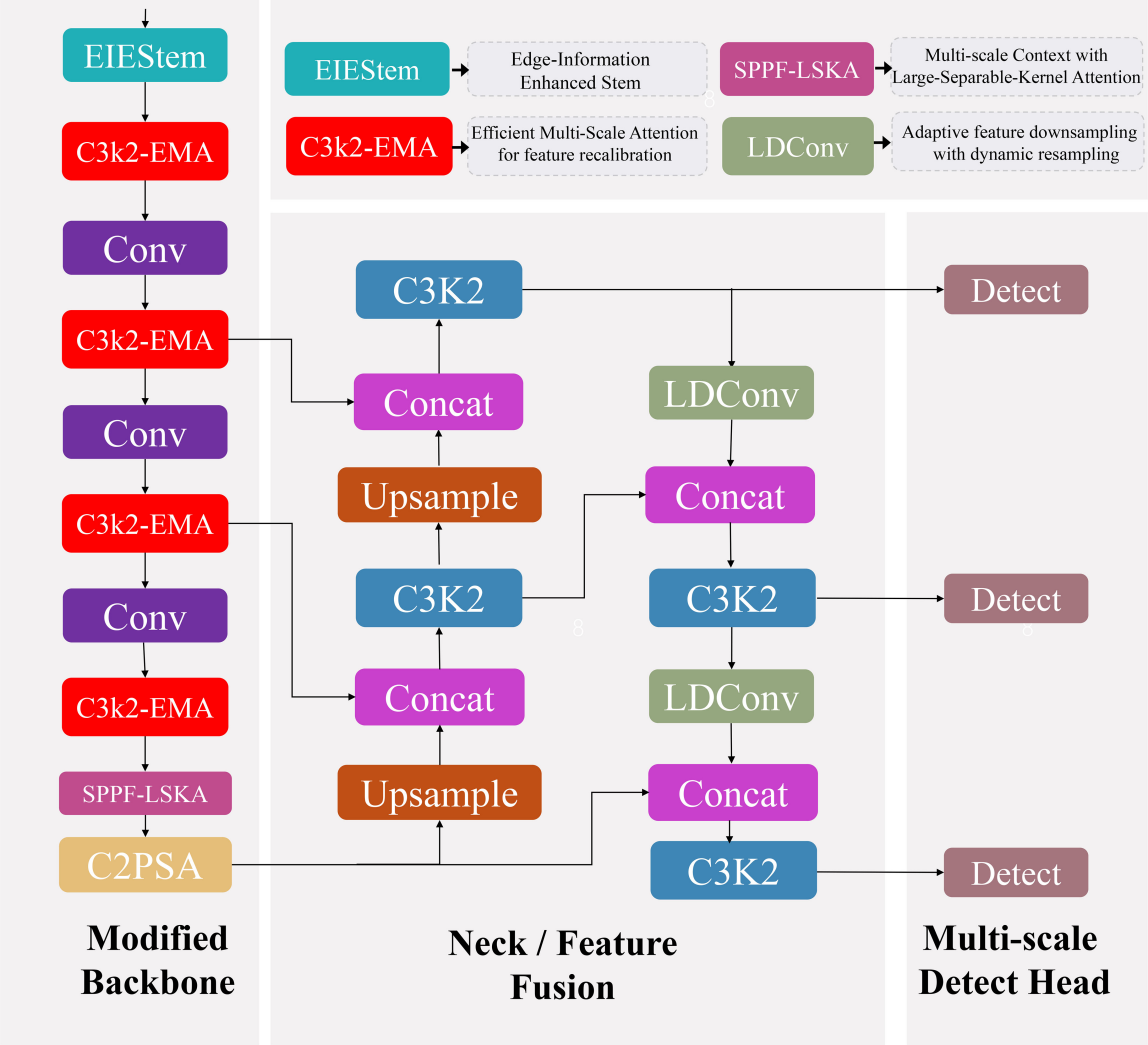
The overall architecture of the improved YOLOv11.

**Figure 2** shows the overall architecture of the improved YOLOv11 (Hidayatullah et al., 2025).

At the front of the backbone, the standard stem block is replaced with EIEStem. This module uses a Sobel-initialized learnable edge branch in the early stage of feature extraction, which helps preserve low-level boundary and texture information while allowing the edge filters to adapt to weed contours in the training data.

In the feature aggregation stage, a C3k2-based feature aggregation module with Efficient Multi-Scale Attention, denoted as C3k2-EMA, is used in the neck. This module improves feature representation and reduces background interference.

At the deepest stage of the backbone, an SPPF-based context enhancement module with Large Separable Kernel Attention, denoted as SPPF-LSKA, is used. This module combines serial pooling with large receptive field attention to strengthen global context modeling.

In the bottom-up fusion path, LDConv is used to replace regular downsampling convolution. By learning adaptive sampling positions, it helps preserve the shape and boundary details of small weed targets during downsampling.

Finally, the fused multi-scale features are fed into three detection heads for small-, medium-, and large-scale weed detection.

Four detector components are placed according to feature hierarchy and the visual characteristics of UAV grassland weeds. EIEStem acts before early information loss occurs, so weak contours and fine edge cues can be retained for later feature fusion. C3k2-EMA then recalibrates mid-level responses across scales, helping the neck highlight weed-like regions while suppressing redundant grassland background. SPPF-LSKA works at a deeper semantic stage, where a larger receptive field is needed to distinguish weeds from visually similar surrounding plants. LDConv is placed in the bottom-up fusion path because downsampling is a main source of shape degradation for small and irregular targets.

This arrangement makes the modules complementary rather than redundant. Boundary cues retained by EIEStem provide more reliable local evidence for the following aggregation stage. C3k2-EMA selects useful multi-scale responses from these features, while SPPF-LSKA adds broader scene context to reduce confusion between weeds and non-target vegetation. LDConv further limits geometric information loss when features are downsampled and fused. The integrated detector therefore follows a boundary–scale– context–geometry design logic, rather than stacking several enhancement modules at a single feature level.

### 3.4 Edge prior enhanced backbone stem design (EIEStem)

**Figure 3:**
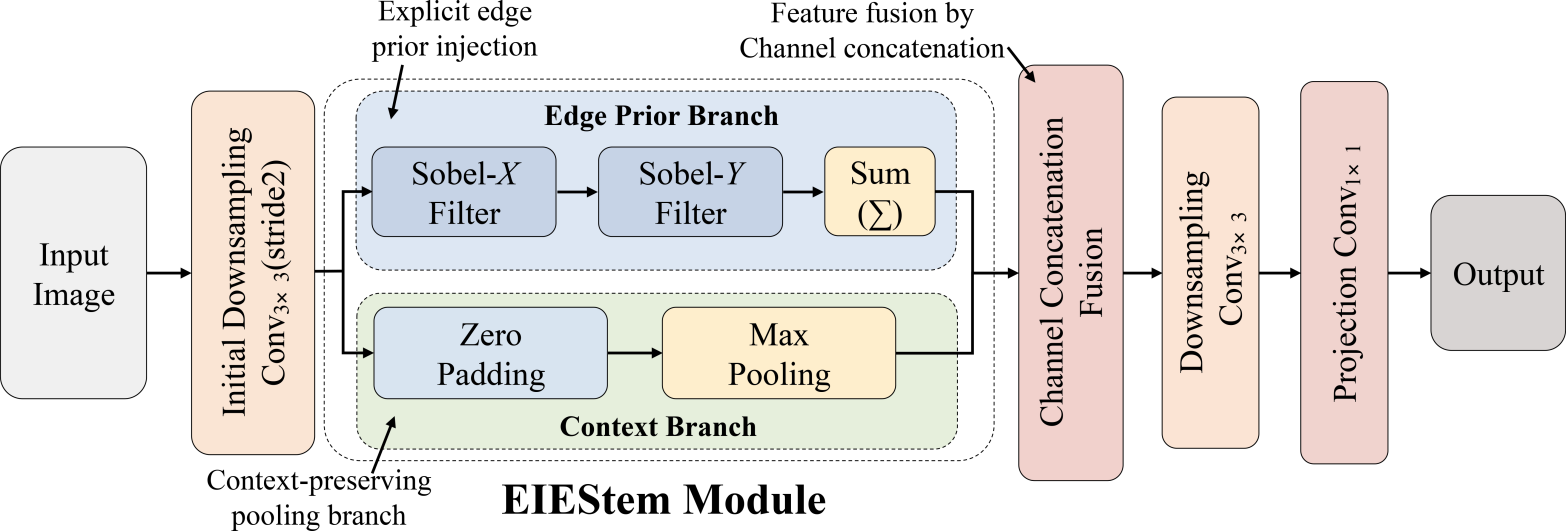
The structure of the EIEStem module used in this study.

In UAV grassland weed detection, weeds often look similar to surrounding grasses and other nontarget vegetation. Key differences mainly appear in subtle texture changes and boundary details. Early downsampling and feature compression in the stem can weaken low-level shape information. An EdgeInformation-Enhanced Stem (EIEStem), shown in **Figure 3**, adds edge information at the front of the backbone and strengthens early feature extraction.

Given an input feature map $X\in {\mathbb{R}}^{H\times W\times C_{in}}$, EIEStem first uses a standard convolution for initial downsampling and channel mapping:


$$X_{s1}=\sigma \left(BN\left(Conv_{3\times 3,\;s=2}\left(X\right)\right)\right)$$

where $X_{s1}\in {\mathbb{R}}^{\frac{H}{2}\times \frac{W}{2}\times C_{hid}}$. The output feature is sent to two parallel branches: an edge branch and a context branch.

An edge branch is used to strengthen boundary information. The horizontal and vertical edge kernels, denoted as $K_{x}\in {\mathbb{R}}^{3\times 3}$ and $K_{y}\in {\mathbb{R}}^{3\times 3}$, are initialized with Sobel operators and then optimized during training. For the *c*-th channel feature map $X_{s1}^{c}$, the edge response *E^c^* is computed as


$$E^{c}=\alpha \cdot \left(K_{x}{⊛}_{dw}X_{s1}^{c}+K_{y}{⊛}_{dw}X_{s1}^{c}\right)$$

where ${⊛}_{dw}$ denotes depthwise convolution, and *α* is a fixed scaling factor used to balance the magnitude of the edge response. In the current implementation, *α* is set to 0.5. This design keeps the gradient prior of Sobel filtering while allowing the edge branch to adapt to the weed-boundary patterns in the training images. Edge feature *X_edge_* is formed by arranging the edge responses from all channels along the channel dimension:


$$X_{edge}={\left[E^{1},E^{2},\ldots ,E^{C_{hid}}\right]}_{C},$$

where [·]*_C_* denotes channel-wise arrangement.

A parallel context branch keeps local information and reduces noise introduced by edge enhancement. Zero padding is first applied to *X_s_*_1_ to preserve the local neighborhood before pooling. Max pooling then produces the context feature:


$$X_{context}=MaxPool_{2\times 2,\;s=1}\left(Pad\left(X_{s1}\right)\right)$$

where *Pad*(·) denotes zero padding.

Then, the outputs of the two branches are concatenated along the channel dimension:


$$X_{fused}=\text{Concat}\left(\left[X_{edge},X_{context}\right],\text{dim}=C\right)$$

A 3 × 3 downsampling convolution followed by a 1 × 1 projection convolution converts the fused feature into the final output $Y_{EIEStem}\in {\mathbb{R}}^{\frac{H}{4}\times \frac{W}{4}\times C_{out}}$:


$$Y_{EIEStem}=\sigma \left(BN\left(Conv_{1\times 1}\left(\sigma \left(BN\left(Conv_{3\times 3,\;s=2}\left(X_{fused}\right)\right)\right)\right)\right)\right)$$

Through this design, EIEStem combines edge enhancement and local information extraction at the early stage of the backbone. It helps the network keep more boundary details and shape information. This is useful for separating weeds from surrounding grassland vegetation with similar appearance.

### 3.5 C3k2-based feature aggregation with Efficient Multi-Scale Attention

**Figure 4:**
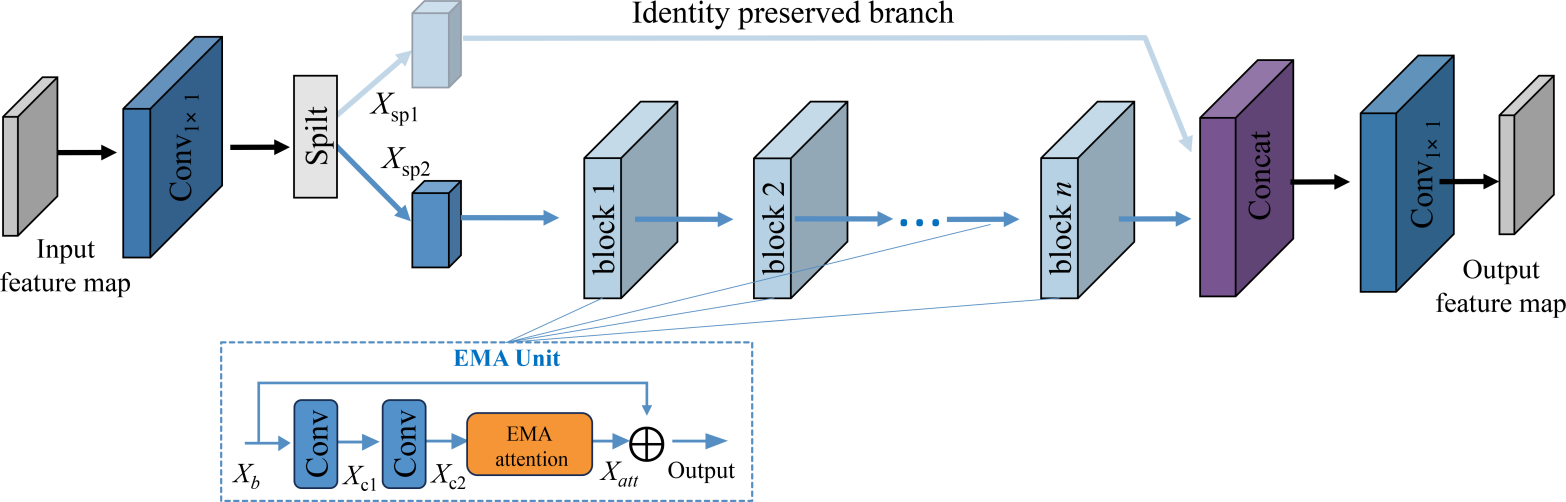
The structure of the integrated C3k2-EMA module.

In weed detection, feature aggregation needs to handle targets with different sizes and complex backgrounds. The original C3k2 block mainly relies on convolution-based feature extraction, while spatially important regions and useful channel responses require additional recalibration. For this reason, a C3k2-based feature aggregation module with Efficient Multi-Scale Attention (EMA), referred to as C3k2-EMA, is used. As shown in **Figure 4**, it combines efficient feature reuse with adaptive attention to improve weed-feature representation.

Given an input feature map $X_{in}\in {\mathbb{R}}^{H\times W\times C_{1}}$, a 1 × 1 convolution is first used for channel projection:


$$\left[X_{sp1},X_{sp2}\right]={\text{Split}}_{1:1}\left(\sigma \left(BN\left(Conv_{cv1}^{1\times 1}\left(X_{in}\right)\right)\right),\text{dim}=C\right)$$

where Split_1:1_ denotes an equal split along the channel dimension. Thus, *X_sp_*_1_ is the identity branch and *X_sp_*_2_ is the enhancement branch, and the two branches have the same number of channels after the 1 × 1 projection. The identity branch keeps the original feature information, whereas the enhancement branch is used for deeper feature refinement.

After splitting, the enhancement branch passes through *n* stacked EMA units in a progressive manner:


$$Z_{0}=X_{sp2},\;Z_{i}=M_{EMA}\left(Z_{i-1}\right),\;i=1,2,\ldots ,n$$

where *Z_n_* is the final enhanced feature.

Each *M_EMA_* unit follows a bottleneck-style design. Given an input feature $X_{b}\in {\mathbb{R}}^{H\times W\times c}$, two consecutive 3 × 3 convolutions are first used to extract local information:


$$X_{c1}=\sigma \left(BN\left(Conv_{3\times 3}\left(X_{b}\right)\right)\right)$$

$$X_{c2}=\sigma \left(BN\left(Conv_{3\times 3}\left(X_{c1}\right)\right)\right)$$

These operations help strengthen local textures, edges, and shape patterns, which are important for weed detection.

After that, EMA is applied to recalibrate the extracted feature:


$$X_{att}=\text{EMA}\left(X_{c2}\right)$$

EMA processes features in groups to keep the computation efficient. It uses parallel operations to capture both local information and broader spatial context. In this way, the module can highlight important weed regions and suppress irrelevant background responses.

A residual connection is then introduced to preserve the original information and stabilize training:


$$M_{EMA}\left(X_{b}\right)=X_{b}+X_{att}$$

After the stacked enhancement units, the identity branch and the enhanced branch are fused by concatenation and a 1 × 1 convolution:


$$X_{out}=Conv_{cv2}^{1\times 1}\left(\text{Concat}\left(X_{sp1},Z_{n}\right)\right)$$

Through this design, C3k2-EMA combines feature reuse, local feature extraction, and adaptive attention in one module. It helps the network focus more effectively on weed-related features. It also reduces the influence of complex agricultural backgrounds.

### 3.6 SPPF-based context enhancement with Large Receptive Field Attention

**Figure 5:**
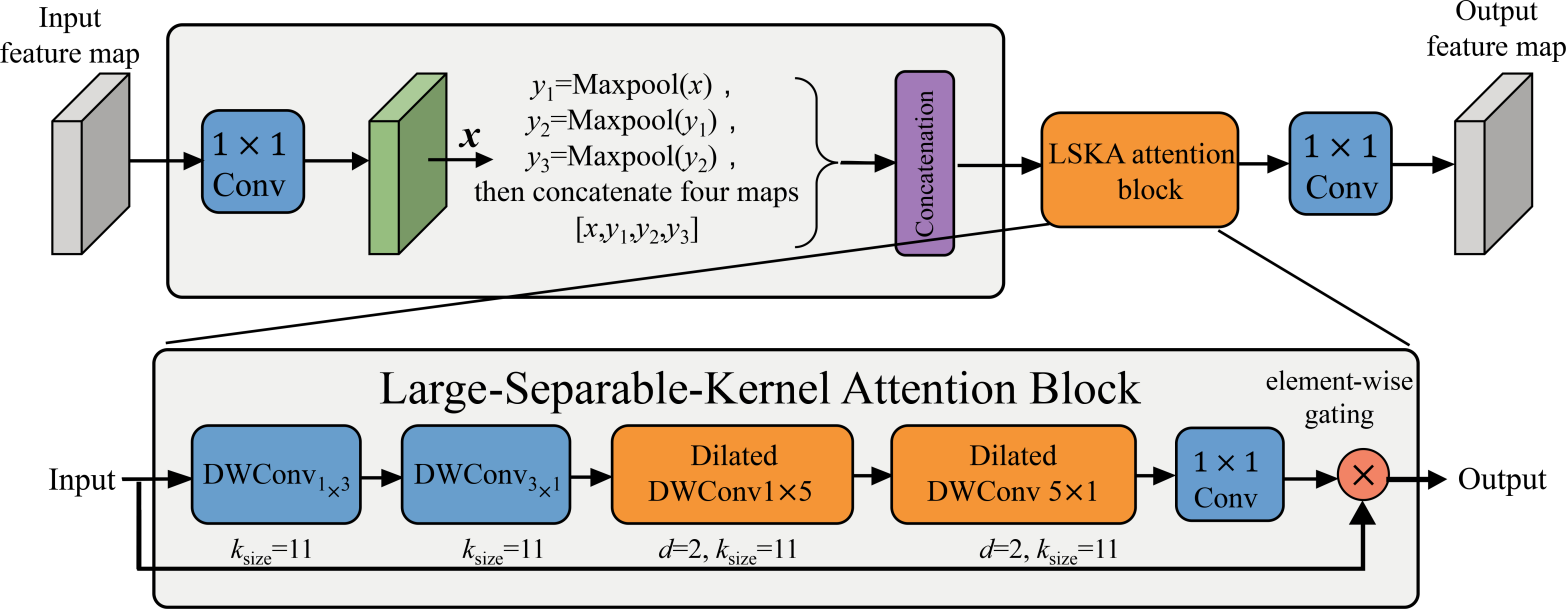
The structure of the integrated SPPF-LSKA module.

In the high-level part of the backbone, simple pooling is often not enough to capture global context. For this reason, an SPPF-based context enhancement module with Large Separable Kernel Attention (LSKA), hereafter referred to as SPPF-LSKA, is integrated into the backbone. **Figure 5** shows the module structure. Multi-scale pooling is combined with large-range spatial attention, allowing a larger spatial range to be modeled while keeping computation efficient.

Given an input feature map *X_in_*, a 1 × 1 convolution is first used to reduce the channel dimension:


$$X_{r}=Conv_{1\times 1}\left(X_{in}\right)$$

where $X_{r}\in {\mathbb{R}}^{H\times W\times C}$.

Then, three max-pooling operations are applied in sequence to extract context information at different scales. Let *Y*_1_, *Y*_2_, and *Y*_3_ denote the outputs of the three pooling operations. These features are concatenated with *X_r_* along the channel dimension:


$$X_{sppf}=\text{Concat}\left(\left[X_{r},Y_{1},Y_{2},Y_{3}\right],\text{dim}=C\right)$$

After that, *X_sppf_* is fed into the LSKA block. The attention branch is set to an effective kernel size of 11 with a dilation rate of 2. This setting is used as a balanced context range for UAV grassland weed features. A smaller spatial range would mainly capture local texture and may miss the surrounding plant layout, whereas a much larger range may introduce more grassland background and increase computation. The dilation rate *d* = 2 expands the receptive field without using a dense large convolution, so the module can model nearby weed–background context while preserving local continuity.

First, two standard depthwise convolutions are used to capture local spatial information:


$$A_{1}={\text{DWConv}}_{1\times 3}^{conv0h}\left(X_{sppf}\right)$$

$$A_{2}={\text{DWConv}}_{3\times 1}^{conv0v}\left(A_{1}\right)$$

Then, two dilated depthwise convolutions are used to further expand the receptive field. The dilation rate is set to *d* = 2:


$$A_{3}={\text{DWConv}}_{1\times 5}^{spatial\_h,\;d=2}\left(A_{2}\right)$$

$$A_{4}={\text{DWConv}}_{5\times 1}^{spatial\_v,\;d=2}\left(A_{3}\right)$$

Next, a 1 × 1 convolution is used to generate the attention map:


$$F_{attn}=\sigma \left(Conv_{1\times 1}^{conv1}\left(A_{4}\right)\right)$$

Multiplying the attention map with the input feature highlights spatially important regions:


$$X_{lska}=X_{sppf}⊙F_{attn}$$

Finally, a projection convolution is used to obtain the output feature:


$$Y=Conv_{cv2}\left(X_{lska}\right)$$

Through this design, SPPF-LSKA combines multi-scale pooling and large-range spatial attention in one module. The 11-size effective kernel provides a broader neighborhood for high-level feature modeling, while *d* = 2 keeps the attention operation sparse enough for lightweight detection. This helps the network distinguish weed regions from visually similar surrounding vegetation in complex grassland scenes.

### 3.7 Adaptive feature downsampling based on Learnable Dynamic Convolution

**Figure 6:**
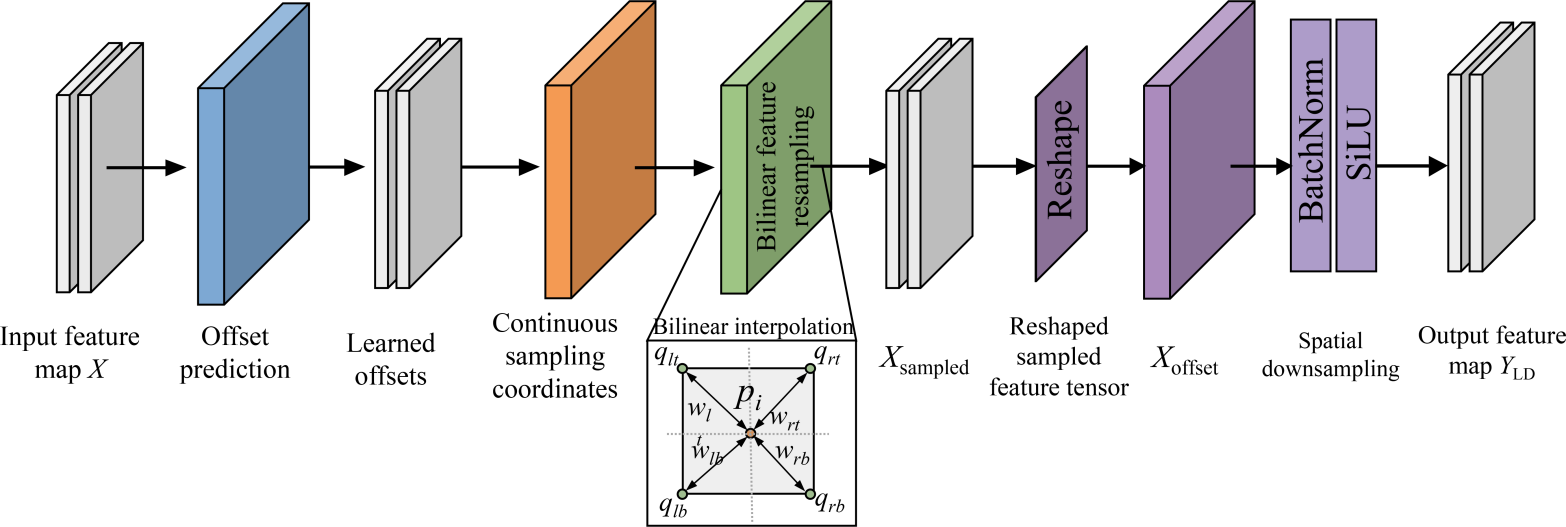
The framework of the LDConv downsampling module used in this study.

Learnable Dynamic Convolution (LDConv) is introduced into the bottom-up fusion path of the Neck network. **Figure 6** shows the LDConv framework. Regular stride-2 convolution uses fixed sampling locations, which can weaken boundary details and local geometric structures during downsampling. LDConv learns adaptive sampling positions from the input feature map and provides a more flexible downsampling operation for irregular weed targets.

Given an input feature map $X\in {\mathbb{R}}^{H\times W\times C_{in}}$, an offset prediction branch *Conv_p_* is first used to estimate the sampling offsets at each spatial position:


$$\text{Δ}P=Conv_{p}\left(X\right)\in {\mathbb{R}}^{H\times W\times 2N}$$

where *N* is the number of sampling points for each position, and 2*N* denotes the horizontal and vertical offsets in the 2D plane.

Based on the regular reference position *p*_0_, the *i*-th dynamic sampling coordinate is defined as


$$p_{i}=p_{0}+\text{Δ}p_{i}$$

where Δ*p_i_* is the learned offset of the *i*-th sampling point. In this way, the sampling process is no longer limited to fixed grid locations. Instead, it can adapt to the spatial distribution of weed features.

Since *p_i_* is usually a continuous coordinate, it cannot be directly indexed on the discrete feature map. Therefore, bilinear interpolation is used to obtain the feature response at each sampling point. The sampled feature at *p_i_* is computed as


$$X_{sampled}\left(p_{i}\right)=\sum_{q}G\left(q,p_{i}\right)\cdot X\left(q\right)$$

where *q* denotes the neighboring integer grid points around *p_i_*, *X*(*q*) is the feature value at grid point *q*, and *G*(*q,p_i_*) is the bilinear interpolation weight. The interpolation kernel is defined as


$$G\left(q,p_{i}\right)=\text{max }(0,1-\left|q_{x}-p_{ix}\right|)\cdot \text{max }(0,1-\left|q_{y}-p_{iy}\right|)$$

After bilinear interpolation, the sampled features from all dynamic sampling points are arranged along the sampling-point dimension to form *X_offset_*:


$$X_{offset}={\left[X_{sampled}\left(p_{1}\right),X_{sampled}\left(p_{2}\right),\ldots ,X_{sampled}\left(p_{N}\right)\right]}_{N},$$

where [·]*_N_* denotes arrangement along the dynamic sampling-point dimension. The sampled features are then reshaped. A convolution with kernel size (*N*,1) and stride (*N*,1) is applied, followed by batch normalization and SiLU activation, to complete feature aggregation and spatial downsampling:


$$Y_{LD}=\text{SiLU}\left(\text{BN}\left(Conv_{N\times 1,\;s=\left(N,1\right)}\left(\text{Reshape}\left(X_{offset}\right)\right)\right)\right)$$

Output channels of *Y_LD_* follow the output channel setting of *Conv_N_*_×1_ and match the corresponding neck feature-fusion stage in YOLOv11.

Through this design, LDConv enables the downsampling operation to adapt to irregular target structures. It helps preserve boundary information and local geometric details during feature compression. This is beneficial for weed detection in complex agricultural scenes.

## 4 Results and discussion

All experiments follow a unified setting. The section begins with dataset partitioning, annotation configuration, and label-quality analysis, then reports module-level and design-level ablations. Comparisons with mainstream detectors and qualitative visualization are presented afterward.

### 4.1 Experimental environment and evaluation metrics

#### 4.1.1 Hardware and software environment

All experiments used the same hardware and software environment. **Table 2** summarizes the computing platform, software framework, and acceleration environment for the ablation and comparison experiments.

**Table 2:** Experimental configuration.

Item	Configuration
Operating system	Ubuntu 22.04 LTS (64-bit)
CPU	Intel Xeon Gold 6230R
GPU	NVIDIA GeForce RTX 3090 (24 GB)
Deep learning framework	PyTorch 2.0.0 and Torchvision 0.15.0
CUDA/cuDNN	CUDA 11.8 and cuDNN 8.7.0
Programming language	Python 3.9
Implementation basis	YOLOv11 training framework

#### 4.1.2 Dataset construction and division

Dataset construction follows the annotation workflow described in Section 3.2. SAM provides instance masks, SigLIP performs few-shot semantic matching, and a VLM audits ambiguous candidates. **Table 3** summarizes the annotation settings. The resulting dataset contains 2,360 UAV grassland images and 8,742 weed bounding boxes. For detector evaluation, the images are divided into five image-level folds. In each run, four folds are used for training and the remaining fold is used for validation. The reported scores are the mean ± standard deviation over the five folds.

**Table 3:** Detailed configuration of the SAM–SigLIP–VLM annotation workflow.

Component	Setting	Purpose
SAM model	SAM ViT-H in automatic mask generation mode.	Generates instance-level vegetation candidate masks from UAV images.
Mask filtering	minimum mask area = 64 pixels, stability score *>* 0.85, and mask NMS IoU = 0.70.	Removes extremely small, unstable, or duplicated vegetation masks.
SigLIP backbone	SigLIP-B/16 with 384 × 384 input resolution.	Encodes candidate patches and few-shot weed references into a shared image-text embedding space.
Similarity metric	Cosine similarity.	Measures semantic similarity between each candidate and the weed reference library.
SigLIP threshold	cosine similarity threshold *τ* = 0.35.	Retains weed-like candidates and filters visually similar background vegetation.
Few-shot support set	10 representative weed patches selected from the training images.	Used by SigLIP for similarity matching and by the VLM as visual reference examples.
VLM model	Qwen3-VL-32B, locally deployed.	Audits ambiguous candidates using few-shot support patches, the candidate patch, and the full UAV image.
VLM system prompt	*You are a UAV grassland weed annotation assistant. Inputs are: (1) ten weed support patches, (2) one local candidate patch, and (3) the full UAV image. Decide whether the candidate is a weed instance. Use the support patches for visual comparison and the full image for scene and boundary context. Return: label = weed/nonweed/uncertain; confidence = 0–1; reason = one short sentence.*	Guides Qwen3-VL-32B to make a weed/non-weed decision and route uncertain cases to manual verification.
Human verification	two annotators checked ambiguous candidates and corrected false positives, missed objects, and inaccurate boxes.	Checks difficult cases and corrects false positives, missing objects, and inaccurate boxes.
Reference annotation	200 reference images, two independent annotators, and third-annotator review.	Used for annotation-quality assessment.
YOLO conversion	Retained mask candidates are converted into normalized bounding boxes.	Provides YOLO-format labels for detector training.

Annotation efficiency and label quality are compared across several annotation strategies using candidate recall, label precision, quality-check error rate, and time cost. Candidate recall denotes the proportion of reference weed instances recalled by the automatic candidate generation stage. Label precision denotes the proportion of generated labels that are correct after comparison with the reference annotations, where both category correctness and bounding-box consistency are considered. The quality-check error rate denotes the proportion of generated annotations that do not match the reference labels in a sampled quality audit, including false positives, missing instances, inaccurate boxes, and wrong semantic judgments. Time cost is normalized by fully manual annotation, which is set to 100%. **Table 4** summarizes the results.

**Table 4:** Annotation quality, agreement, and efficiency comparison.

Annotation strategy	Reference images	Candidate recall	Label precision	Mean IoU	F1-score	Time/image
Fully manual annotation	200	–	–	–	–	96.8 s
SAM only + verification	200	0.913	0.821	0.734	0.865	52.4 s
SAM + SigLIP + verification	200	0.896	0.884	0.779	0.890	39.2 s
SAM + SigLIP + VLM + verification	200	0.887	0.924	0.813	0.905	31.6 s

For this evaluation, 200 images are randomly selected and manually annotated in YOLO format. Two trained annotators label weed bounding boxes from the original images. Inconsistent boxes and missed instances are reviewed by a third annotator to obtain the final reference labels. Candidate recall, label precision, mean IoU, and F1-score are computed using these labels. Average verification time is recorded on the same image subset for each annotation strategy.

As shown in **Table 4**, the SAM–SigLIP–VLM-assisted workflow improves label precision, mean IoU, and F1-score compared with the SAM-only and SAM–SigLIP variants, while requiring less verification time than fully manual annotation. For object-detection annotation, mean IoU, precision, recall-related measures, and F1-score directly measure the agreement between generated boxes and manually reviewed reference boxes.

#### 4.1.3 Model training hyperparameters

Detector experiments share the same training settings, and **Table 5** lists the corresponding hyperparameters.

**Table 5:** Model training hyperparameters.

Item	Setting
Baseline detector	YOLOv11
Input image size	640 × 640
Training epochs	100
Batch size	16
Optimizer	SGD
Momentum	0.937
Initial learning rate	0.01
Learning-rate schedule	cosine decay with 3 warm-up epochs
Weight decay	0.0005
Data augmentation	Mosaic (enabled), horizontal flip (*p* = 0.5), and HSV augmentation (*h* = 0.015, *s* = 0.70, *v* = 0.40)
Evaluation metrics	Precision, Recall, mAP@0.5, and mAP@0.5:0.95

#### 4.1.4 Evaluation metrics

Detection performance is evaluated using standard object detection metrics. Here, TP denotes correctly detected weed targets, FP denotes background regions incorrectly predicted as weeds, and FN denotes missed weed targets. Precision (*P*) and Recall (*R*) are defined as


$$P=\frac{TP}{TP+FP}$$

$$R=\frac{TP}{TP+FN}$$

Average Precision (AP) is defined as the area under the Precision-Recall curve:


$$AP=\int_{0}^{1}P\left(R\right)\;dR$$

Mean Average Precision (mAP) is the average AP over all classes. If *C* is the number of classes, then mAP is defined as


$$mAP=\frac{1}{C}\sum_{i=1}^{C}AP_{i}$$

Here, mAP@0.5 denotes mAP at an IoU threshold of 0.5 and reflects basic detection performance. mAP@0.5:0.95 averages mAP over IoU thresholds from 0.5 to 0.95, providing a stricter evaluation of localization quality.

### 4.2 Downstream impact of assisted annotation

A downstream comparison is conducted to examine whether labels produced by the SAM–SigLIP–VLM workflow can support detector training. A fixed train–test split is used for this experiment rather than the five-fold evaluation protocol. The training images are annotated in two ways: fully manual annotation and SAM–SigLIP–VLM-assisted annotation followed by human verification. Two YOLOv11 baselines with the same architecture, input resolution, and training schedule are then trained separately on these two training-label sets. Both models are evaluated on the same held-out test images, whose labels are fully manual annotations. In this way, the comparison reflects the effect of the training-label source while keeping the test annotations unchanged.

**Table 6** shows that the two training-label sources lead to close results on the same manually annotated test set. The detector trained with SAM–SigLIP–VLM-assisted labels obtains a mAP@0.5 of 0.722, compared with 0.725 for the detector trained with fully manual labels. This small gap indicates that the assisted labels retain similar downstream training utility while reducing the verification time reported in **Table 4**.

**Table 6:** Downstream detector performance under different training-label sources.

Training-label source	Detector	Precision	Recall	mAP@0.5	mAP@ 0.5:0.95
Fully manual annotation	YOLOv11	0.858	0.680	0.725	0.501
SAM–SigLIP–VLM + verification	YOLOv11	0.854	0.676	0.722	0.498
Difference	–	−0.004	−0.004	−0.003	−0.003

### 4.3 Core modules ablation study

Progressive module ablation uses the original YOLOv11 as the baseline. Each component is added step by step, and performance is reported as mean ± standard deviation across five folds. Parameter number and GFLOPs describe the corresponding accuracy-complexity trade-off.

**Table 7** shows a steady performance gain as the four components are introduced. Compared with the YOLOv11 baseline, the proposed model raises mAP@0.5 from 0.722 to 0.762 and mAP@0.5:0.95 from 0.498 to 0.545. This gain comes with a moderate increase in Params, from 2.59M to 3.42M, and GFLOPs, from 6.50 to 7.60.

**Table 7:** Progressive ablation study of the introduced modules.

Model variant	EIE Stem	C3k@-EMA	SPPF-LSKA	LDConv	Params/M	GFLOPs	Precision	Recall	mAP@0.5	mAP@ 0.5:0.95
YOLOv11 baseline	–	–	–	–	2.59	6.50	0.854 ± 0.006	0.676 ± 0.008	0.722 ± 0.006	0.498 ± 0.007
+ EIEStem	✓	–	–	–	2.59	6.68	0.866 ± 0.005	0.684 ± 0.007	0.734 ± 0.005	0.516 ± 0.006
+ C3k2-EMA	✓	✓	–	–	2.60	7.08	0.878 ± 0.005	0.695 ± 0.006	0.748 ± 0.005	0.523 ± 0.006
+ SPPF-LSKA	✓	✓	✓	–	2.87	7.36	0.886 ± 0.004	0.703 ± 0.006	0.755 ± 0.004	0.533 ± 0.005
Proposed model	✓	✓	✓	✓	3.42	7.60	0.895 ± 0.004	0.713 ± 0.005	0.762 ± 0.004	0.545 ± 0.005

### 4.4 EIEStem design ablation study

A controlled branch ablation compares the original stem, a version without an explicit edge prior, a fixed Sobel branch, and a learnable edge branch. This experiment separates two questions: whether edge information is useful in the stem stage, and whether the edge operator should remain fixed or be updated with the detector during training. The detailed EIEStem branch ablation results are summarized in **Table 8**.

**Table 8:** EIEStem branch design ablation.

Variant	Edge branch	Precision	Recall	mAP@0.5	mAP@ 0.5:0.95
Baseline stem	–	0.854 ± 0.006	0.676 ± 0.008	0.722 ± 0.006	0.498 ± 0.007
EIEStem without edge branch	No edge prior	0.860 ± 0.006	0.681 ± 0.007	0.729 ± 0.006	0.508 ± 0.006
EIEStem with fixed Sobel kernel	Fixed Sobel	0.862 ± 0.006	0.682 ± 0.007	0.731 ± 0.006	0.511 ± 0.006
EIEStem with learnable edge kernel	Learnable	0.866 ± 0.005	0.684 ± 0.007	0.734 ± 0.005	0.516 ± 0.006

The results show that edge information is useful for early feature extraction. Removing the edge prior already improves the baseline stem, and adding a fixed Sobel branch brings a further gain by introducing directional gradient cues. However, weed boundaries in UAV grassland images are often weak, curved, fragmented by occlusion, and mixed with grass texture. Fixed Sobel kernels can capture generic horizontal and vertical gradients, but they cannot adjust to these data-specific boundary patterns. The learnable edge branch starts from Sobel-initialized directional priors and is then optimized with the detector, allowing it to respond better to curved weed contours, small leaves, and weak plant–background transitions. Therefore, the final EIEStem uses Sobel-initialized learnable edge kernels.

### 4.5 C3k2-EMA module placement ablation study

Three C3k2-EMA placements are compared in the YOLOv11 architecture: Backbone only, Neck only, and Backbone + Neck. Results are reported as mean ± standard deviation.

The neck in YOLOv11 is responsible for fusing features from different resolutions before prediction. Placing C3k2-EMA in this part of the network allows the fused *P*_3_, *P*_4_, and *P*_5_ features to be recalibrated toward weed-related responses, which is useful for targets that vary in size and are mixed with grassland background. As shown in **Table 9**, the neck-only setting gives the best overall scores among the tested placements. When C3k2-EMA is inserted only in the backbone, it works before multi-scale fusion and has less direct effect on cross-scale target aggregation. When it is used in both the backbone and neck, repeated attention recalibration may weaken part of the fine local cues needed for small and ambiguous weeds. Therefore, the final detector places C3k2-EMA in the neck.

**Table 9:** C3k2-EMA placement ablation.

Placement	Precision	Recall	mAP@0.5	mAP@ 0.5:0.95
Backbone only	0.872 ± 0.006	0.689 ± 0.007	0.742 ± 0.006	0.519 ± 0.006
Neck only	0.878 ± 0.005	0.695 ± 0.006	0.748 ± 0.005	0.523 ± 0.006
Backbone + Neck	0.875 ± 0.006	0.692 ± 0.007	0.744 ± 0.006	0.521 ± 0.006

### 4.6 LDConv design ablation study

LDConv is evaluated by disabling or simplifying its internal components, allowing the effects of adaptive sampling and offset refinement on irregular weed localization to be examined. The detailed LDConv design ablation results are summarized in **Table 10**.

**Table 10:** LDConv design ablation.

Variant	Offset learning	Adaptive sampling	Precision	Recall	mAP@0.5	mAP@0.5:0.95
Standard downsampling	–	–	0.886 ± 0.004	0.703 ± 0.006	0.755 ± 0.004	0.533 ± 0.005
LDConv without offset refinement	Partial	✓	0.889 ± 0.005	0.706 ± 0.006	0.757 ± 0.005	0.537 ± 0.005
LDConv without adaptive sampling	✓	Partial	0.891 ± 0.004	0.708 ± 0.006	0.759 ± 0.004	0.540 ± 0.005

Full LDConv achieves the best overall performance among the compared variants. Offset refinement and adaptive sampling together improve localization, although several variants remain close in score.

### 4.7 Comparison of different feature downsampling mechanisms

**Table 11** compares LDConv with standard convolution, WaveletPool, SPD-Conv, ADConv, DCNv2, and DCNv3 under the same detector setting.

**Table 11:** Comparison of different feature downsampling mechanisms.

Downsampling module	Precision	Recall	mAP@0.5	mAP@ 0.5:0.95
Standard Conv	0.886 ± 0.004	0.703 ± 0.006	0.755 ± 0.004	0.533 ± 0.005
WaveletPool	0.887 ± 0.005	0.705 ± 0.006	0.756 ± 0.005	0.536 ± 0.006
SPD-Conv	0.889 ± 0.005	0.707 ± 0.006	0.758 ± 0.005	0.538 ± 0.006
ADConv	0.891 ± 0.004	0.709 ± 0.006	0.759 ± 0.004	0.540 ± 0.005
DCNv2	0.892 ± 0.005	0.710 ± 0.006	0.760 ± 0.005	0.541 ± 0.006
DCNv3	0.893 ± 0.005	0.711 ± 0.006	0.761 ± 0.005	0.543 ± 0.006
LDConv	0.895 ± 0.004	0.713 ± 0.005	0.762 ± 0.004	0.545 ± 0.005

Among the tested downsampling mechanisms, LDConv obtains the highest precision, recall, and mAP values, indicating that adaptive sampling is more suitable for irregular weed instances than fixed downsampling.

### 4.8 Comparison with state-of-the-art models

**Table 12** compares the proposed detector with YOLOv11–YOLOv13 baselines, transformer-based RTDETR detectors, and recent YOLO-based weed-detection models. Params and GFLOPs are retained for this full-detector comparison to show the overall accuracy-complexity trade-off.

**Table 12:** Comparison with state-of-the-art detection models.

Model	Params/M	GFLOPs	Precision	Recall	mAP@0.5	mAP@0.5:0.95
YOLOv11 baseline	2.59	6.50	0.854 ± 0.006	0.676 ± 0.008	0.722 ± 0.006	0.498 ± 0.007
YOLOv12	2.66	6.55	0.856 ± 0.006	0.674 ± 0.008	0.721 ± 0.006	0.497 ± 0.007
YOLOv13	2.70	6.68	0.855 ± 0.006	0.673 ± 0.008	0.720 ± 0.006	0.496 ± 0.007
RT-DETR-R18 (Transformer)	20.00	60.70	0.842 ± 0.007	0.659 ± 0.009	0.707 ± 0.008	0.481 ± 0.009
RT-DETR-R50 (Transformer)	42.00	136.00	0.851 ± 0.007	0.666 ± 0.008	0.715 ± 0.007	0.489 ± 0.008
GTDR-YOLOv12 [Yang et al., 2025]	2.23	4.80	0.864 ± 0.006	0.682 ± 0.007	0.731 ± 0.006	0.508 ± 0.007
AGRI-YOLO [Peng et al., 2025]	1.38	3.20	0.861 ± 0.006	0.681 ± 0.007	0.729 ± 0.006	0.506 ± 0.007
Proposed model	3.42	7.60	0.895 ± 0.004	0.713 ± 0.005	0.762 ± 0.004	0.545 ± 0.005

All competing models were evaluated under the same detection protocol, including input resolution, validation split, epoch number, optimizer, learning-rate schedule, and augmentation strategy. RT-DETRR18 and RT-DETR-R50 are included as transformer-based detector baselines, while GTDR-YOLOv12 and AGRI-YOLO provide comparison with recent YOLO-based weed-specific adaptations. Although GTDR-YOLOv12 and AGRI-YOLO have fewer parameters and lower GFLOPs, the proposed detector achieves higher detection accuracy on the evaluated weed-detection task. This result indicates that the proposed design does not pursue the smallest model size alone. Instead, it aims to improve detection reliability for UAV grassland weeds by jointly enhancing boundary representation, multi-scale feature fusion, contextual perception, and irregular-structure preservation. The increase in model complexity remains moderate compared with transformer-based detectors such as RT-DETR-R18 and RT-DETR-R50. The complexity values of GTDR-YOLOv12 and AGRI-YOLO follow their original papers, while those of the YOLOv11 baseline and the proposed detector are measured from local model summaries.

### 4.9 Visual comparison of detection results before and after improvement

**Figure 7:**
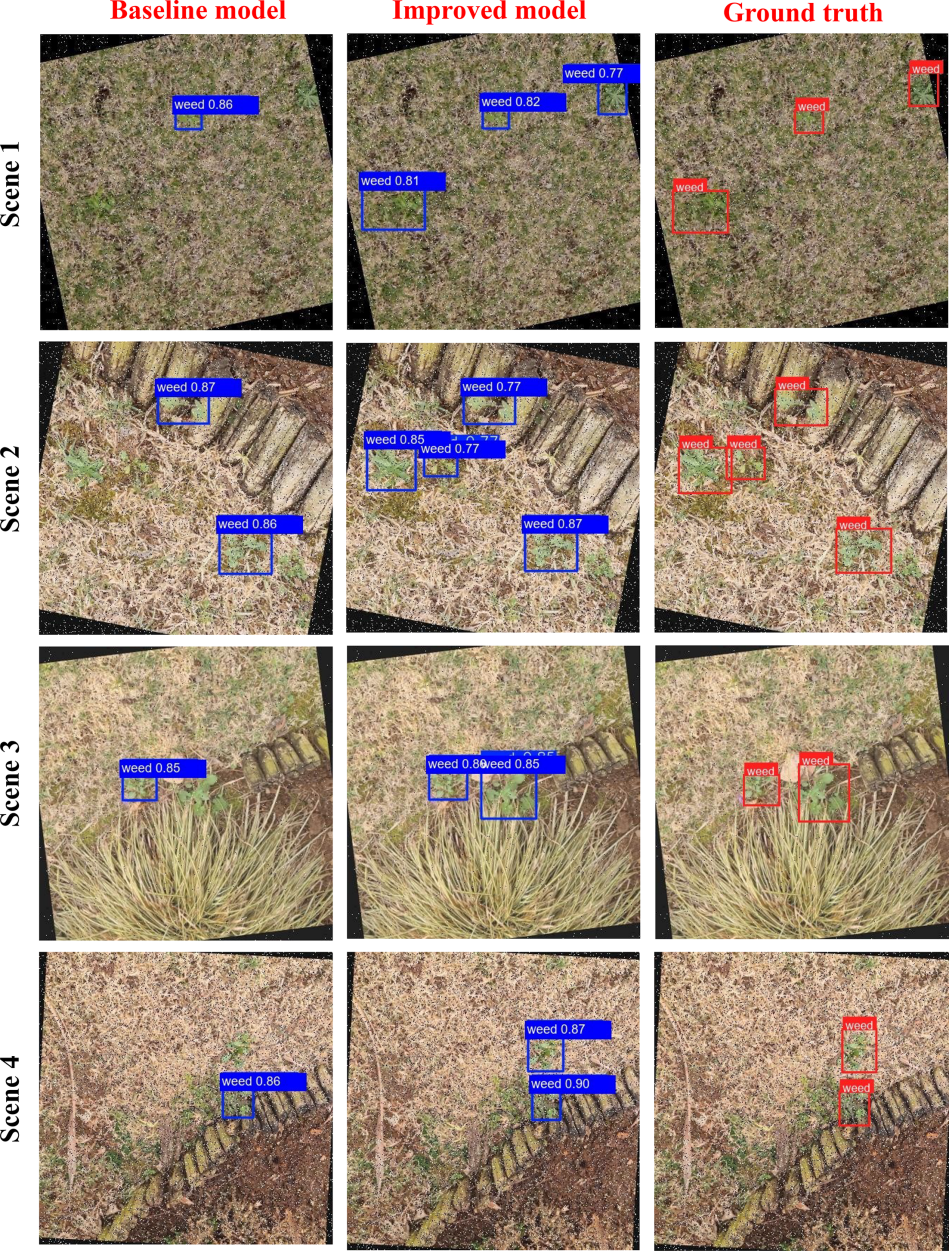
Qualitative comparison of single-class weed detection results.

**Figure 7** compares the YOLOv11 baseline and the proposed model in representative grassland scenes. In visually complex backgrounds, the baseline model mainly suffers from missed detections when weed targets have irregular shapes, weak boundaries, or high similarity to surrounding vegetation. By contrast, the proposed model detects more weed instances in these examples and keeps the predicted boxes closer to the target regions. This qualitative improvement agrees with the design roles of the integrated components: EIEStem preserves shallow boundary information, C3k2-EMA enhances discriminative feature responses, SPPF-LSKA strengthens contextual modeling, and LDConv improves the retention of fine geometric structures during downsampling.

### 4.10 External dataset ablation on CropAndWeed

CropAndWeed is used as an external public crop–weed detection dataset for the additional ablation study. Following the same progressive module-integration strategy as **Table 7**, EIEStem, C3k2-EMA, SPPF-LSKA, and LDConv are introduced into the YOLOv11 baseline step by step. The detailed ablation results on the CropAndWeed dataset are reported in **Table 13**.

**Table 13:** Ablation study on the CropAndWeed dataset.

Model variant	EIE Stem	C3k2 -EMA	SPPF -LSKA	LDConv	Precision	Recall	mAP@0.5	mAP@0.5:0.95
YOLOv11 baseline	–	–	–	–	0.812 ± 0.009	0.641 ± 0.010	0.684 ± 0.008	0.431 ± 0.009
+ EIEStem	✓	–	–	–	0.823 ± 0.008	0.650 ± 0.009	0.695 ± 0.007	0.445 ± 0.008
+ C3k2-EMA	✓	✓	–	–	0.835 ± 0.008	0.662 ± 0.009	0.707 ± 0.007	0.456 ± 0.008
+ SPPF-LSKA	✓	✓	✓	–	0.843 ± 0.007	0.671 ± 0.008	0.716 ± 0.006	0.466 ± 0.007
Proposed model	✓	✓	✓	✓	0.856 ± 0.006	0.684 ± 0.007	0.728 ± 0.006	0.479 ± 0.007

Results on CropAndWeed show the same progressive improvement trend. The full model achieves higher precision, recall, mAP@0.5, and mAP@0.5:0.95 than the YOLOv11 baseline on this external dataset.

### 4.11 General discussion and limitations

The detector design follows a complementary feature-enhancement chain rather than a single isolated modification. EIEStem strengthens shallow boundary cues for weak weed contours, C3k2-EMA recalibrates multi-scale features in the neck, SPPF-LSKA expands contextual perception for weed–background discrimination, and LDConv preserves irregular geometry during downsampling. These components act on different stages of feature propagation, so their combination is consistent with the visual difficulties of UAV grassland weed detection, including small targets, ambiguous boundaries, mixed vegetation, and irregular plant shapes.

The framework also combines annotation assistance with detector adaptation. SAM generates candidate masks, SigLIP performs few-shot weed-like matching, and Qwen3-VL-32B audits ambiguous candidates using support patches, local candidate crops, and full-image context. The downstream annotation-source comparison indicates that verified SAM–SigLIP–VLM labels can train the same YOLOv11 detector with only a small performance difference from fully manual labels. Nevertheless, dense vegetation, shadow, and partial occlusion still require human verification, and the annotation workflow should be further tested on larger field collections.

Several limitations remain. First, the proposed detector is not the smallest model among the compared methods; GTDR-YOLOv12 and AGRI-YOLO have lower Params and GFLOPs. The present design instead targets higher detection accuracy with moderate complexity. Second, robustness under diverse illumination, occlusion, and weather conditions has not been fully evaluated. Future work will include multi-region, multi-season, and multi-species UAV weed datasets, with systematic tests under strong shadow, rainfall, low-light conditions, and large seasonal changes.

## 5 Conclusions

A UAV-based weed detection framework for grassland scenes was developed by combining annotation assistance with task-oriented detector adaptation. At the data level, SAM, SigLIP, and a vision-language model generate instance candidates, perform few-shot semantic matching, and audit ambiguous weed-like samples with local and contextual information. Human verification is retained for ambiguous cases.

At the model level, an improved YOLOv11-based detector was constructed by integrating EIEStem, C3k2EMA, SPPF-LSKA, and LDConv. These components enhance boundary preservation, feature aggregation, contextual modeling, and adaptive downsampling for irregular weed targets. Under five-fold image-level cross-validation, the proposed model achieved 0.762 ± 0.004 mAP@0.5 and 0.545 ± 0.005 mAP@0.5:0.95, improving the YOLOv11 baseline by 4.0 and 4.7 percentage points, respectively. Additional evaluation on CropAndWeed showed a similar improvement trend, suggesting that the proposed design can be transferred to another single-class weed detection setting.

## Data Availability

The raw data supporting the conclusions of this article will be made available by the authors, without undue reservation.

## References

[B1] Arshad M. A. Jubery T. Z. Roy T. Nassiri R. Singh A. K. Singh A. . (2025). “ Leveraging vision language models for specialized agricultural tasks”, in: Proceedings of the IEEE/CVF Winter Conference on Applications of Computer Vision (WACV), 6320–6329.

[B2] Belissent N. Peña J. M. Mesías-Ruiz G. A. Shawe-Taylor J. Pérez-Ortiz M. (2024). Transfer and zero-shot learning for scalable weed detection and classification in UAV images. Knowledge-Based Syst. 292, 111586. doi: 10.1016/j.knosys.2024.111586 38826717

[B3] Bhandari U. Burlakoti S. Miller R. Young S. Westra E. Etienne A. (2026). USU-Corn-WeedDB: A UAV RGB image dataset for multi-species weed detection in forage corn. arXiv. doi: 10.48550/arXiv.2606.06709. Preprint.

[B4] Chinnasami R. K. Rajappan S. Murugasamy V. (2025). UAV-based intelligent weed detection using YOLO11 and PSPNet for precision agriculture. Notulae Botanicae Horti Agrobotanici Cluj-Napoca 53, 14822. doi: 10.15835/nbha53414822

[B5] Gerhards R. Andújar Sanchez D. Hamouz P. Peteinatos G. G. Christensen S. Fernandez-Quintanilla C. (2022). Advances in site-specific weed management in agriculture–a review. Weed Res. 62, 123–133. doi: 10.1111/wre.12526 40046247

[B6] Hidayatullah P. Syakrani N. Sholahuddin M. R. Gelar T. Tubagus R. (2025). YOLOv8 to YOLO11: A comprehensive architecture in-depth comparative review. arXiv. 10(2), 341–354. doi: 10.48550/arXiv.2501.13400. Preprint.

[B7] Kamilaris A. Prenafeta-Boldú F. X. (2018). Deep learning in agriculture: A survey. Comput. Electron. Agric. 147, 70–90. doi: 10.1016/j.compag.2018.02.016 38826717

[B8] Kirillov A. Mintun E. Ravi N. Mao H. Rolland C. Gustafson L. . (2023). “ Segment anything”, in: Proceedings of the IEEE/CVF International Conference on Computer Vision (ICCV), 3992–4003. doi: 10.1109/ICCV51070.2023.00371

[B9] Krestenitis M. Raptis E. K. Kapoutsis A. C. Ioannidis K. Kosmatopoulos E. B. Vrochidis S. . (2022). CoFly-WeedDB: A UAV image dataset for weed detection and species identification. Data Brief 45, 108575. doi: 10.1016/j.dib.2022.108575 36131952 PMC9483728

[B10] Li J. Li D. Savarese S. Hoi S. C. H. (2023). “ BLIP-2: Bootstrapping language-image pre-training with frozen image encoders and large language models Proceedings of Machine Learning Research”, Honolulu, Hawaii: PMLR in: Proceedings of the 40th International Conference on Machine Learning, 202, 19730–19742.

[B11] Liu Y. Zeng F. Diao H. Zhu J. Ji D. Liao X. . (2024). YOLOv8 model for weed detection in wheat fields based on a visual converter and multi-scale feature fusion. Sensors 24, 4379. doi: 10.3390/s24134379 39001158 PMC11244458

[B12] Mu Y. Feng R. Ni R. Li J. Chen Y. Liu W. (2022). A Faster R-CNN-based model for the identification of weed seedling in images of cropping areas. Agronomy 12, 2867. doi: 10.3390/agronomy12112867 30654563

[B13] Nasir M. F. Rehman M. U. Hussain I. (2026). Vision-language models for zero-shot weed detection and visual reasoning in UAV-based precision agriculture. Front. Plant Sci. 16, 1735096. doi: 10.3389/fpls.2025.1735096 41695537 PMC12894358

[B14] Olsen A. Konovalov D. A. Philippa B. Ridd P. Wood J. C. Johns J. . (2019). DeepWeeds: A multiclass weed species image dataset for deep learning. Sci. Rep. 9, 2058. doi: 10.1038/s41598-018-38343-3 30765729 PMC6375952

[B15] Peng G. Wang K. Ma J. Cui B. Wang D. (2025). AGRI-YOLO: A lightweight model for corn weed detection with enhanced YOLOv11n. Agriculture 15, 1971. doi: 10.3390/agriculture15181971 30654563

[B16] Rahman A. Lu Y. Wang H. (2023). Performance evaluation of deep learning object detectors for weed detection for cotton. Smart Agric. Technol. 3, 100126. doi: 10.1016/j.atech.2022.100126 38826717

[B17] Redmon J. Divvala S. Girshick R. Farhadi A. (2016). “ You only look once: Unified, real-time object detection”, in: Proceedings of the IEEE Conference on Computer Vision and Pattern Recognition (CVPR), 779–788. doi: 10.1109/CVPR.2016.91

[B18] Sa I. Popović M. Khanna R. Chen Z. Lottes P. Liebisch F. . (2018). WeedMap: A large-scale semantic weed mapping framework using aerial multispectral imaging and deep neural network for precision farming. Remote Sens. 10, 1423. doi: 10.3390/rs10091423 30654563

[B19] Saleem M. H. Potgieter J. Arif K. M. (2022). Weed detection by Faster RCNN model: An enhanced anchor box approach. Agronomy 12, 1580. doi: 10.3390/agronomy12071580 30654563

[B20] Sandoval-Pillajo L. García-Santillán I. Pusdá-Chulde M. Giret A. (2025). Weed detection based on deep learning from UAV imagery: A review. Smart Agric. Technol. 12, 101147. doi: 10.1016/j.atech.2025.101147 38826717

[B21] Steininger D. Trondl A. Croonen G. Simon J. Widhalm V. (2023). “ The CropAndWeed dataset: A multi-modal learning approach for efficient crop and weed manipulation”, in: Proceedings of the IEEE/CVF Winter Conference on Applications of Computer Vision (WACV), 3729–3738. doi: 10.1109/WACV56688.2023.00372

[B22] Yang Z. Khan Z. Shen Y. Liu H. (2025). GTDR-YOLOv12: Optimizing YOLO for efficient and accurate weed detection in agriculture. Agronomy 15, 1824. doi: 10.3390/agronomy15081824 30654563

[B23] Yu G.-H. Anh L. H. Vu D. T. Lee J. Rahman Z. U. Lee H.-Z. . (2025). VL-PAW: A vision– language dataset for pear, apple and weed. Electronics 14, 2087. doi: 10.3390/electronics14102087 30654563

[B24] Yue Y. Zhao A. (2026). Weed segmentation in soybean fields and variable-rate herbicide prescription map generation based on UAV imagery and improved YOLOv11-seg. Front. Plant Sci. 16, 1743263. doi: 10.3389/fpls.2025.1743263 41742959 PMC12929378

[B25] Zhai X. Mustafa B. Kolesnikov A. Beyer L. (2023). “ Sigmoid loss for language image pre-training”, in: Proceedings of the IEEE/CVF International Conference on Computer Vision (ICCV), 11941–11952. doi: 10.1109/ICCV51070.2023.01100

